# Charge and adsorption height dependence of the self-metalation of porphyrins on ultrathin MgO(001) films†

**DOI:** 10.1039/d2cp04688a

**Published:** 2022-11-17

**Authors:** Francesco Presel, Christian S. Kern, Thomas G. Boné, Florian Schwarz, Peter Puschnig, Michael G. Ramsey, Martin Sterrer

**Affiliations:** Institute of Physics, University of Graz, NAWI Graz, Universitätsplatz 5 A-8010 Graz Austria martin.sterrer@uni-graz.at

## Abstract

We have experimentally determined the adsorption structure, charge state, and metalation state of porphin, the fundamental building block of porphyrins, on ultrathin Ag(001)-supported MgO(001) films by scanning tunneling microscopy and photoemission spectroscopy, supported by calculations based on density functional theory. By tuning the substrate work function to values below and above the critical work function for charging, we succeeded in the preparation of 2H-P monolayers which contain negatively charged and uncharged molecules. It is shown that the porphin molecules self-metalate at room temperature, forming the corresponding Mg–porphin, irrespective of their charge state. This is in contrast to self-metalation of tetraphenyl porphyrin (TPP), which occurs on planar MgO(001) only if the molecules are negatively charged. The different reactivity is explained by the reduced molecule-substrate distance of the planar porphin molecule compared to the bulkier TPP. The results of this study shed light on the mechanism of porphyrin self-metalation on oxides and highlight the role of the adsorption geometry on the chemical reactivity.

## Abstract 2

While the self-metalation of 2H-TPP on MgO(001) thin films is mediated by charge transfer, 2H-P self-metalates irrespective of its charge state. The different reactivity is explained based on the molecule-substrate distance.

## Graphical abstract

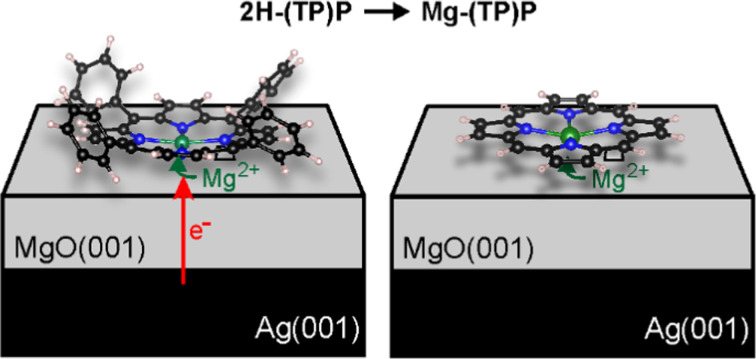

## 1 Introduction

Tailoring the properties of molecules of the tetrapyrrole family by metalation and functionalization is potentially useful for targeting specific applications, *e.g.*, in the fields of catalysis, sensing, and optoelectronics. To this end, on-surface preparation strategies, mostly carried out on metal surfaces, for variously functionalized porphyrins and phthalocyanines have been developed, that provided detailed insight into their hierarchical organization and allowed their structural, electronic and chemical properties to be studied in great detail.^1–3^ However, for specific applications, *e.g.*, if porphyrins are to be used in solar energy harvesting devices,^4^ it is desirable to switch to semiconducting substrates such as oxides. Compared to metal surfaces, atomic-scale investigations into the interfacial properties of hybrid systems oxide/porphyrin are just emerging. Herein, we present a study of the interface between the basic tetrapyrrole macrocycle, the free-base porphin (2H-P), and well-defined MgO(001) surfaces, to elucidate the role of the distance between the macrocycle and the surface, and of the charging of the molecules, on its self-metalation activity.

The controlled synthesis of metal-tetrapyrrole complexes and assemblies is possible by surface-confined methods such as post-metalation or self-metalation of adsorbed molecules.^5^ It is established that free-base porphyrins self-metalate in a redox process on specific metal substrates, *e.g.* Fe, Ni, Co, Pd, Cu, Ag, Au, where it often requires thermal activation or the aid by adsorbed oxygen.^6–11^ Recent studies have also provided insight into the anchoring and self-metalation of porphyrins, specifically of 2H-tetraphenyl-porphyrin (2H-TPP) and its derivatives, on oxide surfaces such as MgO,^12^ TiO_2_,^13^ or cobalt oxides.^14^ In contrast to on metal surfaces, the self-metalation reaction on oxides can be viewed as an ion-exchange process, where the two aminic protons in the macrocycle are replaced by a substrate cation and either desorb, or form hydroxyls on the surface.

2H-TPP adsorbs flat, that is, with the macrocycle parallel to the surface, on most oxide surfaces. Its self-metalation has been shown to depend on the type of oxide. For example, TPP readily metalates on CoO(111) and Co_3_O_4_(111)^9,14^ films at room temperature, but requires thermal activation on TiO_2_(110),^13^ where initially the diacid (4H-TPP) is formed and the metalation process might be triggered by the diffusion of interstitial Ti to the surface.^15–17^ In addition, a strong dependence of the self-metalation activity on oxides on the adsorption geometry has been noted. The introduction of specific anchor groups, *e.g.* carboxylic or phosphonic acid groups attached to the phenyls of TPP, can shift the preferred adsorption geometry from flat-lying to upright standing, which generally leads to suppression of self-metalation, depending on coverage and temperature.^18–27^

While for some flat oxides the high self-metalation yield points to a high activity of regular surface sites in this process, the possible involvement of surface defects has to be considered as well.^28^ A specific case evolved for MgO, for which self-metalation of 2H-TPP was originally demonstrated for MgO nanostructures on the edges of cubic nano-crystals, where the energy needed to extract a magnesium ion is lower and the energy balance due to the formation of hydroxyls is favorable.^29^ Subsequent experiments suggested that also on flat, single-crystalline substrates it only occurs at undercoordinated sites.^12^ In contrast, we have recently shown that it can occur on the regular surface sites of a planar Ag(001)-supported MgO(001) ultrathin film, where the metalation process is facilitated by charge transfer (CT) of electrons from the metal substrate, through the MgO film, into the adsorbed porphyrins.^30^

However, the underlying mechanism allowing or preventing self-metalation based on charge transfer has not been fully understood. One possible explanation is suggested by our previous observation that charge transfer leads to electrostatic attraction, pulling the porphyrin macrocycle of 2H-TPP closer to the MgO surface compared to the uncharged case, where the distance to the surface is larger because of the steric effect of the bulky phenyl ligands.^30^ By similar arguments, the self-metalation activity of 2H-TPP on the edges and corners of nanoparticulate MgO^29^ could be explained by the macrocycle making a closer approach at corners and edges without interference of the steric repulsion of the phenyls.^31^

To provide support for this hypothesis, we present in this work an experimental and computational study about the self-metalation of the free-base porphin (2H-P) on the surface of ultrathin MgO(001) films. Compared to 2H-TPP, the porphin molecule is lacking the four external phenyl ligands and is therefore completely planar, which should allow the macrocycle to get closer to the surface even without the help of electrostatic attraction due to charging. In addition, since the phenyl ligands contribute only little to the frontier molecular orbitals of 2H-TPP, the electronic structures of 2H-TPP and 2H-P in the energy range of interest are almost identical and should therefore not be accountable for any observed differences in self-metalation activity.

Experimentally, we follow a similar approach as previously reported for the study of the self-metalation of 2H-TPP on ultrathin MgO(001) films on Ag(001).^30^ By variation of the work function *Φ* of the MgO(001)/Ag(001) substrate, we are able to control the charge transfer into the 2H-P molecular monolayer and, thus, can study the self-metalation of charged and uncharged molecules. The basic structural characterization of the 2H-P monolayers was performed with scanning tunneling microscopy (STM) and low energy electron diffraction (LEED). The charge and the metalation state of the 2H-P molecules was determined using ultraviolet photoemission spectroscopy (UPS) and X-ray photoemission spectroscopy (XPS), respectively. The result of these measurements, that the self-metalation of 2H-P on ultrathin Ag(001)-supported MgO(001) films does not depend on the charge state of the molecules, is supported by calculations based on density functional theory (DFT).

## 2 Methods

### 2.1 Experimental

The experiments were performed in two separate ultrahigh vacuum apparatuses, one specifically designed for low-temperature scanning tunneling microscopy studies, and the other one for photoemission experiments. The Ag(001) crystal was cleaned by repeated sputtering (sample current *I*_S_ = 4 μA, HV = 750 V) and annealing (*T* = 750 K) cycles. The ultrathin MgO films were then grown *via* reactive Mg deposition, using slightly different growth conditions to obtain films with either standard or high-*Φ*.^32^ The growth rate was controlled by the Mg deposition rate, which was calibrated with a quartz microbalance. To obtain a standard-*Φ* film, Mg was evaporated in *p* = 1.0 × 10^−6^ mbar O_2_ onto the sample kept at *T* = 550 K; the O_2_ flow was then promptly switched off together with the Mg flux and the sample was slowly cooled to RT (10 K min^−1^). The high-*Φ* film was obtained likewise, however the O_2_ pressure was slightly higher (*p* = 2.0 × 10^−6^ mbar); moreover, after interrupting the Mg flux the O_2_ flow was left at the same pressure and the sample temperature was firstly kept constant for 10 minutes, then slowly cooled at the same rate always in O_2_ flow, and the gas flow was only switched off once the sample temperature reached below 400 K. 2H-P (95% purity) from Frontier Scientific was used without further purification and was deposited onto the MgO film held at RT from a home-built evaporator with the porphin powder contained in a crucible, which was heated to 430 K for sublimation. The calibration of the deposited amount was again based on the quartz microbalance and the *Φ* behavior during a dosing series was used to determine the dose corresponding to a monolayer (here we define 1 ML as the single layer completion coverage).

UPS measurements were performed using a NanoESCA system by ScientaOmicron, with a custom-designed preparation chamber attached to it, which is equipped with a sputter gun, a heating stage, the Mg (FOCUS EFM 3T) and molecule (resistively heated crucible) evaporators, leak valves and an XPS setup from SPECS (Phoibos 150 analyzer and XR50 Al-Kα source). The sample temperature during all photoemission experiments was room temperature. Ultraviolet He I (*hν* = 21.22 eV) light was produced by a HIS 14 HD excitation source by Focus and reflected onto the surface at an angle of 68° to the surface normal by a toroidal mirror. UPS spectra were collected with a channeltron detector, and the work function *Φ* was determined from the secondary electron cutoff in a sample bias configuration. LEED and STM measurements were carried out in another set-up, also equipped with the necessary sample preparation equipment. Since the electron beam can damage or even destroy molecular overlayer structures, LEED experiments were always performed after STM experiments. For 2H-P on standard-*Φ* samples LEED images of reasonable quality could be obtained, while for 2H-P on high-*Φ* samples the diffuse pattern present disappeared too quickly. Therefore, no LEED images could be obtained for the corresponding preparations. STM images were obtained at *T* = 77 K in a low-temperature STM system from Createc. Electrochemically etched tungsten tips were used, and the bias voltage was applied to the sample.

### 2.2 Theoretical

The geometric and electronic properties of adsorbed (Mg-) porphin molecules were computed in the repeated slab-approach, using 5 layers of Ag plus 2 layers of MgO as a substrate and a minimum of 18 Å vacuum between the slabs. We used the plane-wave code VASP^33–35^ with the projector-augmented wave method^36^ and a dipole-correction in *z*-direction to avoid spurious electric fields. Since systems with organic molecules on dielectric interlayers on metal substrates have proven to be challenging,^30^ we used the explicit van der Waals-functional optb86b-vdW^37,38^ for exchange–correlation effects, including long-range dispersion. All geometries were relaxed to a total energy convergence of 0.001 eV, with the 3 lowest layers of Ag held fixed (lattice constant: 4.092 Å). We sampled the Brillouin Zone with a Monkhorst–Pack^39^ mesh of 4 × 4 × 1 and used a kinetic energy cutoff of 450 eV. For the simulation of the electronic structure, refined settings with 8 × 8 × 1 *k*-point sampling and 500 eV energy cutoff were used.

## 3 Results and discussion

Charge transfer into adsorbates weakly interacting with MgO(001)/Ag(001) can be well described by the parallel plate capacitor model. Within this model, the amount of transferred charges depends on the work function (*Φ*) and on the thickness of the oxide film.^32^ An important property for charge transfer is the pinning work function (*Φ*_pin_), which is determined by the electronic properties of the adsorbate and describes the highest substrate work function, where charging can still be observed. The *Φ*_pin_ for the 2H-P/MgO(001)/Ag(001) system studied here is 3.8 eV and thus similar to the one of 2H-TPP on the same substrate.^30^ For samples with an initial work function before molecule deposition (*Φ*_ini_) smaller than *Φ*_pin_, charge transfer into the molecules will occur, whereas for samples with *Φ*_ini_ > *Φ*_pin_ no charging will occur. In order to investigate the self-metalation of 2H-P molecules on ultrathin MgO(001) films and its dependence on the charge state of 2H-P, we have prepared 2 monolayer (ML) thin MgO(001) films on Ag(001) with different initial work functions: one, with a *Φ*_ini_ < *Φ*_pin_, will be denoted as “standard-*Φ*” and has been obtained following the typical preparation procedure for flat MgO films;^40,41^ the other, with *Φ*_ini_ > *Φ*_pin_, is denoted “high-*Φ*”. The high *Φ* is obtained by treatment of the standard MgO(001)/Ag(001) thin film with oxygen at elevated temperature, which introduces excess O at the MgO/Ag interface.^42^

**Fig. 1:**
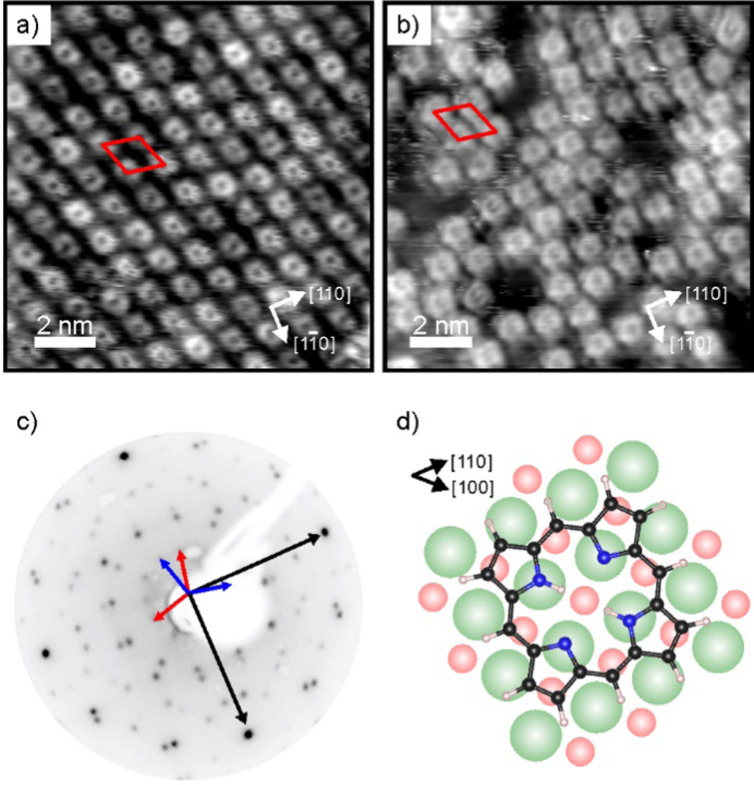
(a and b) STM images (12 nm × 12 nm) of a 2H-P monolayer on (a) standard-*Φ*, and (b) high-*Φ* 2 ML MgO(001)/Ag(001), taken at 77 K. Tunneling conditions: (a) *I*_t_ = 58 pA, *V*_bias_ = +0.1 V; (b) *I*_t_ = 53 pA, *V*_bias_ = +0.39 V. (c) LEED image (55 eV) of 2H-P on standard-*Φ* MgO(001)/Ag(001). The unit cell vectors of the MgO substrate (black) and two mirror domains of the 2H-P superstructure (red and blue) are indicated by arrows. (d) Schematic of the 2H-P adsorption geometry on MgO(001) (green: Mg; red: O; black: C; blue: N; white: H).

As shown by our LEED and STM results reported in Fig. 1, monolayers of 2H-P form a well-ordered overlayer structure on the MgO(001)/Ag(001) substrate. Fig. 1a shows an STM image of 2H-P on the standard-*Φ* sample after deposition at room temperature (RT) and mild heating to 400 K. The corresponding LEED pattern is displayed in Fig. 1c. The lattice formed by the adsorbates corresponds to a commensurate (1,4|4,1) superstructure. Two 90° rotated domains (indicated by the red and blue unit cells in Fig. 1c) are observed in the LEED pattern. In STM, at the shown tunneling conditions, the molecules have a slight rhombic appearance, *i.e.*, with a 2-fold symmetry. It is interesting to note that, although 2H-P forms a supercell not aligned with the high-symmetry directions of the MgO, the symmetry axes of the individual molecules are closely aligned to a 〈100〉 direction of the substrate (as shown in Fig. 1d). In Fig. 1b the STM image of 2H-P on a high-*Φ* sample is shown. The 2H-P coverage here is slightly below full monolayer, and the sample has not been annealed to prevent work function changes due to thermal-induced desorption of oxygen from the MgO/Ag interface. Despite the slightly worse quality of the image, it can clearly be seen that the molecules adsorb with the same orientational alignment as in the standard-*Φ* case and locally arrange in the same superlattice.

**Fig. 2:**
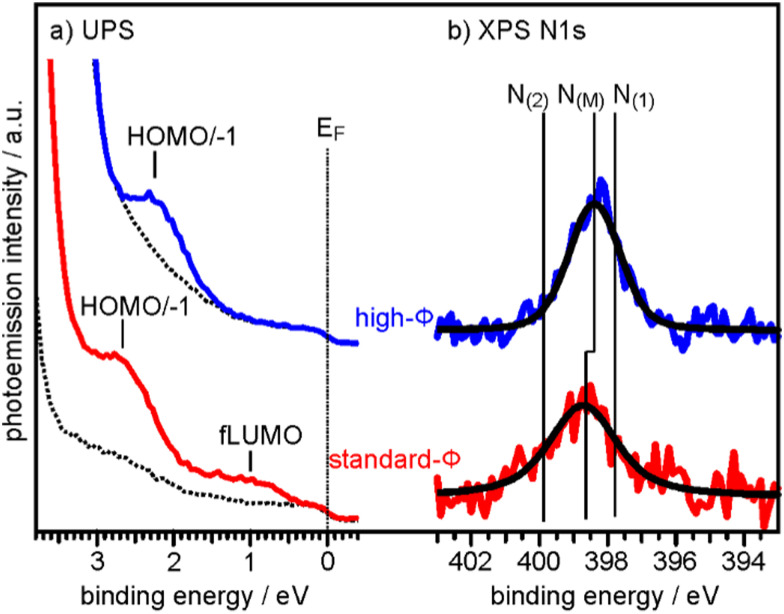
(a) UPS spectra of the clean 2 ML MgO(001)/Ag(001) substrate (broken lines) and of 1 ML 2H-P deposited at RT on a standard-*Φ* (red line, *Φ*_ini_ = 2.73 eV) and a high-*Φ* (blue line, *Φ*_ini_ = 4.08 eV) sample, respectively. The Fermi energy (*E*_F_) and the major emissions corresponding to fLUMO and HOMO/−1 are indicated. (b) N 1s XP spectra of the same sample preparations. Colored lines are raw data and black lines are the corresponding fits assuming only a single N 1s component on each sample. N_(M)_ indicates the BE of the N 1s peaks for the metalated Mg-P, while N_(1)_ and N_(2)_ are the BE's expected for unmetalated 2H-P (see ESI†).

To determine the degree of charge transfer into the 2H-P monolayer on the two substrates, we have measured their ultraviolet photoemission spectra (UPS). The region between the Fermi level (*E*_F_) and the strong MgO valence band (VB) emission differs significantly for the standard-*Φ* and high-*Φ* samples, as can be appreciated in Fig. 2a. For the case of the standard-*Φ* sample, two distinct emissions are detected, at a binding energy (BE) of 2.8 eV and 1.0 eV, respectively. In contrast, on the high-*Φ* sample only a single 2H-P-related emission is apparent at 2.3 eV BE. The UPS spectra obtained here are similar to the ones of 2H-TPP on MgO(001)/Ag(001) thin films of different *Φ*_ini_.^30^ Following similar arguments as in our previous study, the emission at 1.0 eV on the standard-*Φ* sample is assigned to a former lowest unoccupied molecular orbital (fLUMO) of the porphin, which is populated on charging. Note that in an isolated porphin molecule the two lowest unoccupied MO's (LUMO and LUMO+1) are degenerate and no distinction between them is made at this point. The emissions between 1.5 eV and 3 eV on both, the standard and high-*Φ* sample, are a superposition of the 2H-P HOMO and HOMO−1, which are too close in energy to be resolved. Since no molecular emissions appear on the high-*Φ* sample between *E*_F_ and the HOMO/HOMO−1 emissions, we conclude that the 2H-P molecules remain uncharged on this surface. This interpretation is supported by the observation of the work function change upon 2H-P adsorption. While the work function remained constant in the case of the high-*Φ* sample, an increase of about 1 eV was noted for the standard-*Φ* sample, which is consistent with the formation of a charge transfer dipole due to the presence of negatively charged porphin molecules.

To determine if the different charge states of the molecules affect the self-metalation, N 1s XP core level spectra have been acquired from the same samples. As shown in Fig. 2b, the corresponding spectra of 2H-P on high-*Φ* and standard-*Φ* MgO(001)/Ag(001) have a very similar appearance and can both be fitted with a single component, N_(M)_, which is centered at a BE of 398.4 eV for the high-*Φ* sample and at 398.7 eV for the standard-*Φ* sample. The larger width of the spectrum for the standard-*Φ* sample (full-width at half maximum of 2.1 eV compared to 1.6 eV for high-*Φ*) arises from the presence of two species of metalated molecules, namely charged and uncharged ones, which are too close in BE to be resolved. The presence of both charged and uncharged species on an ultrathin MgO(001) film is not unexpected and has, for example, previously been observed within an adsorbed pentacene monolayer.^32^

The appearance of a single component in the N 1s spectrum of porphyrins is the accepted fingerprint of their metalation: unmetalated molecules have two inequivalent nitrogen atoms, as within each molecule two are protonated and two are not, whereas in the metalated case the metal ion is equivalently bonded to all 4 nitrogen atoms, making them equivalent.^43^ For comparison, the XPS of a sample with partially populated second (unmetalated) 2H-P layer is shown in the ESI,† from which we could identify the N 1s BE components originating from unmetalated molecules (N_(1)_ and N_(2)_ in Fig. 2b), separated by 2 eV. In addition, the appearance of a high-BE shoulder in the O 1s XP spectrum after 2H-P deposition, due to the formation of hydroxyl groups, provides further confirmation of the metalation reaction (see ESI†).

From the combined XPS and UPS results we can conclude that the porphin monolayers are fully metalated on, both, high-*Φ* and standard-*Φ* MgO(001)/Ag(001) substrates. Thus, the self-metalation reaction of 2H-P to Mg-P on the planar MgO(001) surface occurs irrespective of the charge state of the molecules. This contrasts with the previously investigated 2H-TPP, which remained unmetalated on a high-*Φ* substrate and was metalated only if charge transfer into the molecules was possible.^30^ Our suggestion that the charging of 2H-TPP is necessary to bring the porphyrin macrocycle closer to the MgO surface, thereby facilitating the self-metalation, is thus strongly supported by the experimental observations.

To corroborate the experimental findings, we performed DFT calculations for a standard-*Φ* (3.1 eV) and a high-*Φ* (4.7 eV) system, respectively. The former is obtained with a stoichiometric MgO(001) film on Ag(001), while the latter is achieved by adding 1/2 ML of oxygen in interstitial sites at the MgO/Ag interface.^32,42^ We have performed the calculations for two unit cell sizes, one resembling the full monolayer (high coverage, HC), and one with a larger unit cell (low coverage, LC) to simulate the situation of a more or less isolated 2H-P(Mg-P) molecule.

**Fig. 3:**
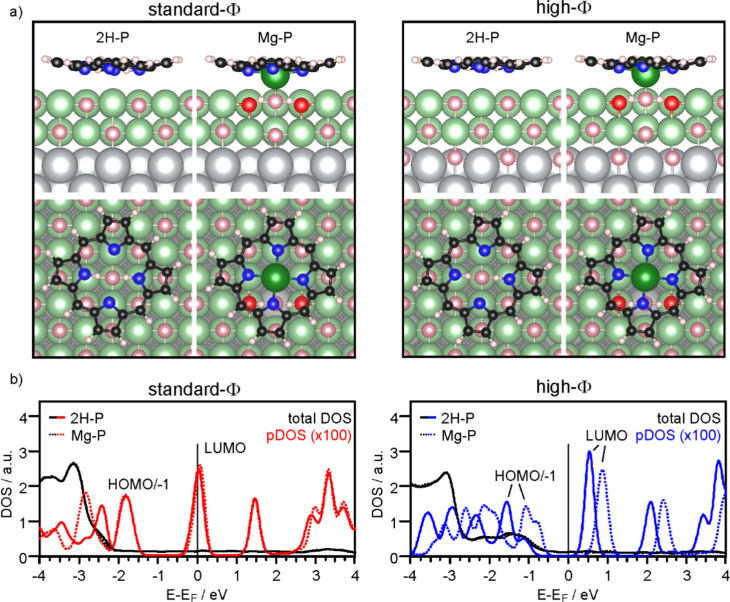
(a) Side views and top views of the DFT (optb86b)-optimized geometry for 2H-P and Mg-P on standard-*Φ* (left panel) and high-*Φ* (right panel) 2 ML MgO(001)/Ag(001). Colors: grey: Ag; pink: O; red: O of OH; green: Mg; black: C; blue: N; white: H. Note that the high *Φ* was obtained by adding 1/2 ML O at interstitial sites of the interfacial Ag layer. (b) Calculated density of states (DOS) for 2H-P (full lines) and Mg-P (dotted lines) on standard-*Φ* (left panel) and high-*Φ* (right panel) 2 ML MgO(001)/Ag(001). The black and colored lines represent the total DOS and the DOS projected onto the C atoms of 2H-P/Mg-P, respectively.

Firstly, we discuss the adsorption configuration (side views and top views in Fig. 3a) for the low coverage case. The adsorption geometry was found to be the same regardless of the work function (Fig. 3, left panel: standard-*Φ*; right panel: high-*Φ*). The inner part of the macrocycle is slightly bent towards the surface with an average height of 2.69 Å and 2.83 Å, respectively (Table 1). The pyrrolic nitrogen atoms are located on top of surface Mg^2+^ ions with the two aminic protons pointing towards a surface O^2−^ ion below the center of the molecule. The calculated density of states (DOS) is shown in Fig. 3b. Here, a significant difference is observed between the two systems: for high *Φ*, the DOS has a clear gap around the Fermi level (*E*_F_), with the LUMO located about 0.5 eV above it, while for standard *Φ* the LUMO crosses *E*_F_. These results agree qualitatively with the experimental data, showing that on the high-*Φ* sample the molecules remain neutral, whereas on the standard-*Φ* sample the molecules get negatively charged.

**Table 1:** Calculated distance d(N-surface) between the average position of the 4 nitrogen atoms in the porphyrin macrocycle and the average position of the Mg ions in the topmost MgO layer, and metalation energy, Δ*E*_met_ (= *E*_Mg-P/MgO(001)/Ag(001)_ − *E*_2H-P/MgO(001)/Ag(001)_), for 2H-P and Mg-P on 2 ML MgO(001)/Ag(001) as a function of the initial work function, *Φ*_ini_. For comparison, the d(N-surface) for 2H-TPP and Mg-TPP on 2 ML MgO(001)/Ag(001) are also shown. Results for 2H-P are reported for calculations employing the optb86b vdW functional for the high-coverage case (HC) and the low-coverage case (LC)

*Φ*_ini_/eV	*d*(N-surface)/Å		Δ*E*_met_/eV	d(N-surface)/Å		Δ*E*_met_/eV	*d*(N-surface)/Å^b^	
	2H-P(LC)	Mg-P(LC)		2H-P(HC)	Mg-P(HC)		2H-TPP	MgTPP
3.1 (Standard-*Φ*)	2.69	2.62	−0.04	3.09^a^	2.74	−0.25	2.74	2.77
4.7 (High-*Φ*)	2.83	2.71	−0.83	3.10^a^	2.74	−0.68	3.08	2.97

a The d(N-surface) of 2H-P provided for the HC case refers to adsorption with the center of the molecule above an Mg ion, which is energetically slightly more favorable than adsorption above an O ion.

b The results for the TPP's were computed with a different treatment of van der Waals forces (see ESI of ref. 30).

The self-metalation was simulated by exchanging an Mg^2+^ ion from the surface with the aminic protons. The subsequent geometry relaxation shows that the protons bind with the O^2−^ in the Mg^2+^ vacancy forming hydroxyls, and that the exchanged Mg^2+^ is positioned slightly below the molecular backbone. As shown in Fig. 3a, the overall adsorption geometry is hardly modified by metalation. For high *Φ*, there is a significant stabilization of the metalated state over the unmetalated one by 0.83 eV, whereas for standard *Φ* the energy difference is small (Table 1). This is most likely related to the fact that the unmetalated 2H-P, due to charging, adopts already an energetically favorable adsorption height for metalation. Indeed, on the standard-*Φ* surface the *N*-surface distance is only marginally reduced upon metalation (Table 1). Regardless of the work function, the Mg^2+^ ion in the macrocycle (Mg_p_) is coordinated to a surface O^2−^, which is significantly lifted from the surface plane (by 0.25 Å). The Mg_P_–O_surf_ distance is 2.06 Å, which is only slightly larger than the Mg–O distance in the film (2.04 Å in our calculation) and shows that in the metalated case, the extracted Mg^2+^ ion adopts a position that resembles the continuation of the Mg–O lattice in vertical direction. Qualitatively, the DOS of the metalated molecules shows a similar charging behavior to that observed for 2H-P, with the LUMO crossing *E*_F_ in the case of standard *Φ*, and a clear HOMO–LUMO gap around *E*_F_ for high *Φ* (Fig. 3b).

To investigate the role of lateral interactions between molecules in the adsorbed ML, we repeated all calculations with a smaller unit cell (corresponding to the experimental ML). The adsorption geometry and DOS obtained for these systems is shown in the ESI.† Most significantly, the favorable adsorption site for the unmetalated molecules is with their center above a surface Mg^2+^ ion, and they are further away from the surface than at lower coverage: the d(N-surface) increases from 2.75 Å to about 3.1 Å for, both, high-*Φ* and standard-*Φ*. By contrast, for Mg-P a similar geometry and molecule-to-surface heights were obtained as in the low coverage case (Table 1). Also the DOS shows some differences at quantitative level, but the qualitative behavior is the same (see ESI†). For the high-*Φ*, the LUMO shifts up in energy. For standard-*Φ*, a similar upward shift results in a smaller area of the LUMO DOS being below *E*_F_, representing a smaller CT. This is expected based on the capacitor model, as the same charge must be distributed between more molecules to reach the same pinning work function. The smaller CT can, however, not account for the increased d(N-surface) alone. This is clear by comparing the d(N-surface) of the high and low coverage cases on the high-*Φ* system, where no charge transfer takes place. The difference of 0.27 Å (Table 1) results primarily from intermolecular interactions. Finally, also for the full ML case, metalation is, at least thermodynamically, still favored, with a calculated energy gain of 0.25 eV (standard-*Φ*) and 0.68 eV (high-*Φ*), respectively.

It is instructive to compare the N-surface heights for the 2H-P/Mg-P systems with those of TPP, where metalation was strongly dependent on the charge state. For standard-*Φ*, where significant CT into TPP takes place, the d(N-surface) is about 2.75 Å for 2H-TPP and Mg-TPP, whereas for high-*Φ*, where no CT takes place and no metalation was observed in experiment, it is around 3 Å (Table 1). By contrast, for 2H-P in the larger unit cell, the dependence of d(N-surface) on *Φ* and thus CT is much weaker, with values of 2.62 Å (standard-*Φ*) and 2.73 Å (high-*Φ*), respectively. This shows that in uncharged 2H-P, when isolated on the surface, the center of the macrocycle can indeed approach closer to the surface than in uncharged 2H-TPP, which substantiates our conclusion about the critical role of the distance between the macrocycle and the surface for the self-metalation. Care has to be taken, however, when comparing the results on oxides with those for the self-metalation on metal surfaces, where molecular hydrogen is formed as byproduct of the redox reaction. For example, the self-metalation of 2H-TPP and 2H-P on copper surfaces requires elevated temperature, even though both molecules receive a significant amount of charge on copper and the d(N-surface) is only 2.2 Å.^44–46^ On the other hand, the process proceeds readily at room temperature upon water formation in the presence of additional oxygen,^47^ which highlights the decisive role of the reaction pathway and the involved energetics.

## 4 Conclusions

Our experimental data, supported by DFT calculations, clearly shows that charge transfer in the 2H-P/MgO(001)/Ag(001) system is strongly affected by the work function of the MgO(001)/Ag(001) substrate. By tuning the preparation conditions, we were able to prepare 2H-P monolayer films, which are either uncharged or charged, similar as previously reported for 2H-TPP on the same substrate.^30^ Compared to the latter, however, the metalation behavior is remarkably different. Whereas 2H-TPPs were only able to self-metalate when charge transfer occurred, a 2H-P monolayer can self-metalate completely, regardless of whether the molecules are charged or not. This provides an important insight into the self-metalation process, as it proves that charge transfer does not play a direct role in this reaction. Our results suggest instead that the key factor enabling it is the distance between the macrocycle and the surface. In the case of the bulkier 2H-TPP, electrostatic attraction induced by charging provides the force that pulls the macrocycle so close to the surface that self-metalation is facilitated. On the other hand, the planar 2H-P can reach this critical distance without the help of charging.

Since a MgO(001)/Ag(001) thin film substrate with a high work function can be considered as bulk-like MgO in terms of its charge transfer properties, the results presented in this work suggest that the self-metalation of 2H-P on planar MgO(001) faces is not restricted to thin film substrates, but should be observable also on the (001) facets of, *e.g.* MgO nanocubes, where the self-metalation of 2H-TPP is not possible.^29^

## Conflicts of interest

There are no conflicts to declare.

## Supplementary Material

CP-024-D2CP04688A-s001
