# Supplementary material for: Charge and adsorption height dependence of the self-metalation of porphyrins on ultrathin MgO(001) films
Source: Phys Chem Chem Phys. 2022 Nov 17;24(46):28540–7. doi: 10.1039/d2cp04688a (PMC9710497; doi:10.1039/d2cp04688a)
Supplement: CP-024-D2CP04688A-s001 [file CP-024-D2CP04688A-s001.pdf]

Electronic Supplementary Material (ESI) for Physical Chemistry Chemical Physics.
This journal is © the Owner Societies 2022

Supporting Information for

Charge and adsorption height dependence of the self-metalation of porphyrins on ultrathin MgO(001) films

Francesco Presel,[1] Christian S. Kern,[1] Thomas G. Boné,[1] Florian Schwarz,[1] Peter Puschnig,[1] Michael G. Ramsey,[1] Martin Sterrer[1]

[1]Institute of Physics, NAWI Graz, University of Graz, Universitätsplatz 1, 8010 Graz, Austria.

SI1: XPS of a partially populated second 2H-P layer

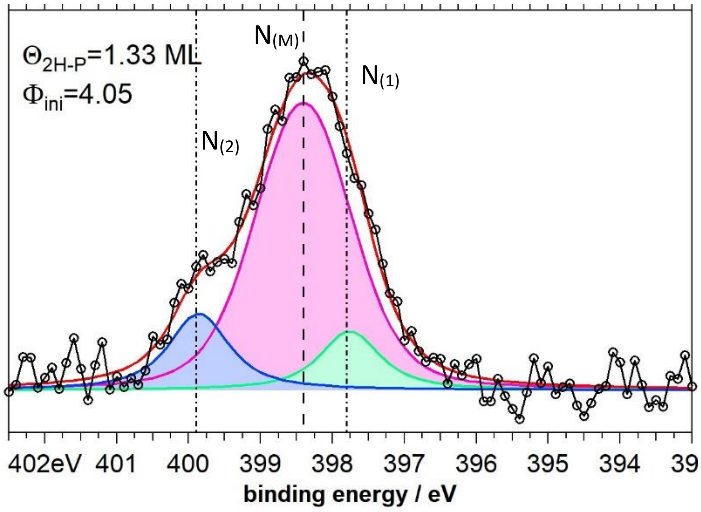

Figure SI1. XP spectrum of 1.33 ML 2H-P on high-Φ 2 ML MgO(001)/Ag(001). The spectrum has been fitted with three peaks, corresponding to the 4 equivalent N atoms in metalated MgP ($N_{(M)}$, BE = 398.4 eV)) and the iminic ($N_{(1)}$, BE = 397.8 eV) and aminic ($N_{(2)}$, BE = 399.8 eV) N atoms in unmetalated 2H-P, which grows on top of the metalated monolayer.

SI2: XPS of O 1s region before and after 2H-P deposition

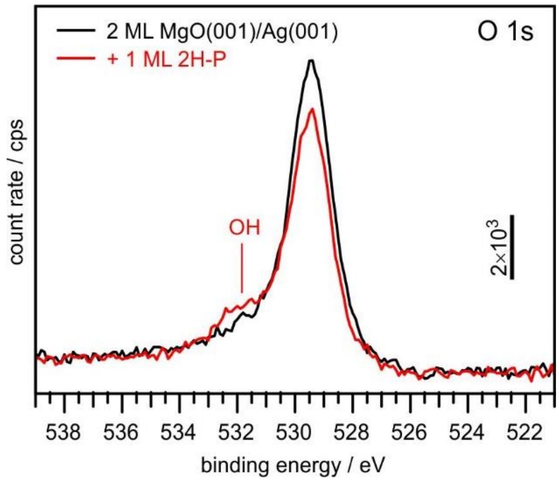

Figure SI2. O 1s XP spectrum of clean 2 ML MgO(001)/Ag(001) (black) and of the same surface after deposition of 1 ML 2H-P (red). In addition to the attenuation of the main O 1s signal from the MgO film (529.5 eV), a shoulder grows in at higher binding energy (532 eV), which is associated with the formation of hydroxyl groups. [SI1]

SI3: Computational results for 2H-P and Mg-P at high coverage

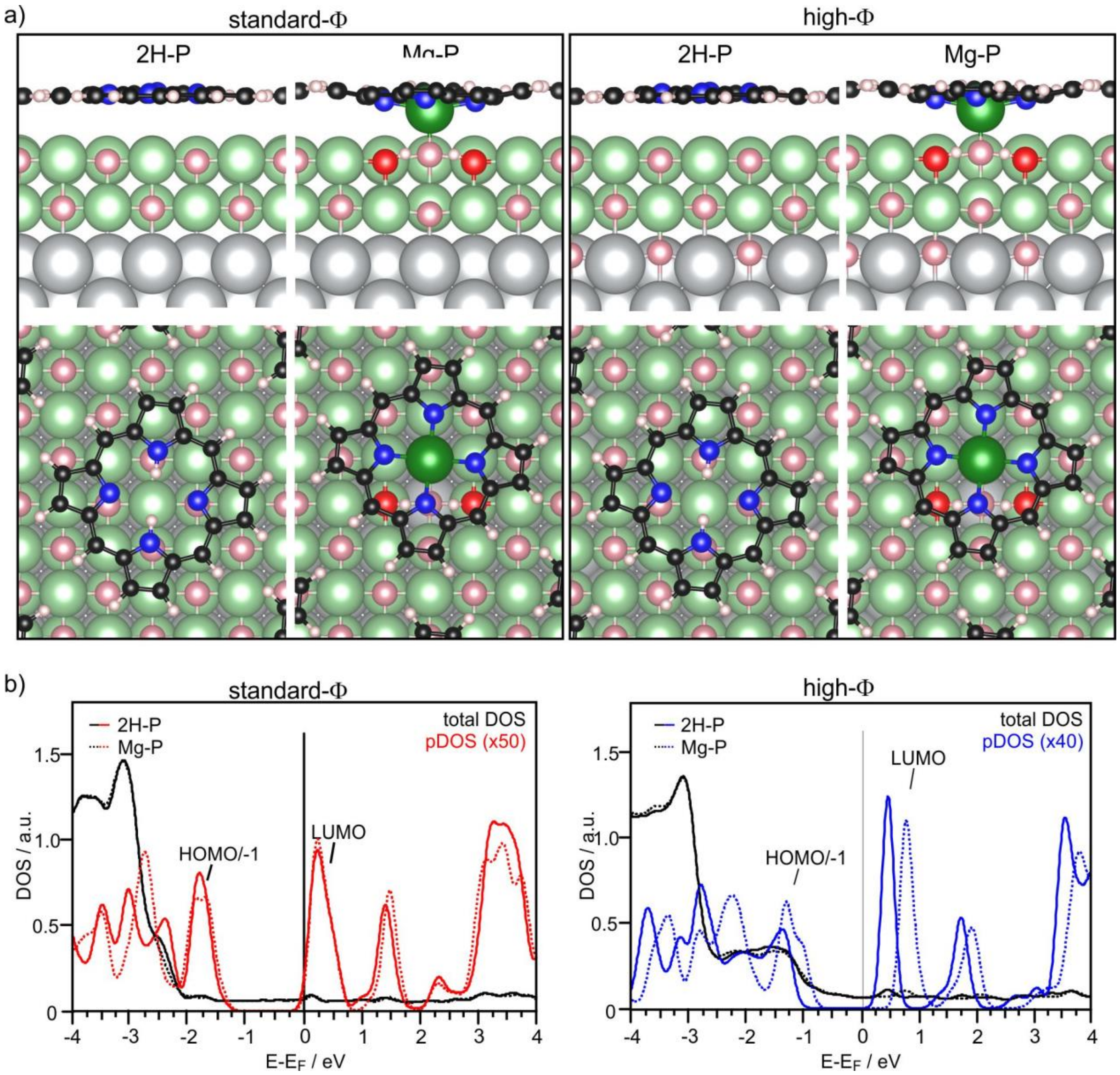

Figure SI3. a) Side views and top views of the DFT (optb86b)-optimized geometry for 2H-P and Mg-P on standard-Φ (left panel) and high-Φ (right panel) 2 ML MgO(001)/Ag(001). Colors: grey: Ag; pink: O; red: O of OH; green: Mg; black: C; blue: N; white: H. Note that the high Φ was obtained by adding ¼ ML O at interstitial sites of the interfacial Ag layer. b) Calculated density of states (DOS) for 2H-P (full lines) and Mg-P (dotted lines) on standard-Φ (left panel) and high-Φ (right panel) 2 ML MgO(001)/Ag(001). The black and colored lines represent the total DOS and the DOS projected onto the C atoms of 2H-P/Mg-P (enhanced by a factor of 50), respectively.

References

[SI1] G. Di Filippo, A. Classen, R. Pöschel and Thomas Fauster, *J. Chem. Phys.*, 2017, **146**, 064702.
